# Recent Advances in the Diagnosis and Treatment of Vestibular Disorders

**DOI:** 10.3390/jcm12165281

**Published:** 2023-08-14

**Authors:** Nicolas Pérez-Fernández, Angel Ramos-Macías

**Affiliations:** 1 Department of Otorhinolaryngology, Clinica Universidad de Navarra, 28047 Madrid, Spain; 2 Department of Otolaryngology, Head and Neck Surgery, Complejo Hospitalario Universitario Insular Materno Infantil de Gran Canaria, 35001 Las Palmas, Spain; ramosorl@idecnet.com

Vestibular medicine “embraces a wide approach to the potential causes of vestibular symptoms, acknowledging that vertigo, dizziness, and unsteadiness are non-specific symptoms that may arise from a broad spectrum of disorders, spanning from the inner ear to the brainstem, cerebellum and supratentorial cerebral networks, to many disorders beyond these structures” [1]. In recent years, we have seen major changes in the definition and availability of new guidelines for different disorders, a new hierarchy for vestibular examination that begins with vestibular reflex activity evaluation and scales up to vestibular cognition, and the clear assessment of treatment results based on medication, surgery, and vestibular rehabilitation. This framework allows for the correct consideration of new findings from genetics, imaging, and sociodemographic studies. Most of these issues are the topics of the manuscripts in this special collection.

Vestibular implants mark one of the most recent advances in the treatment of patients with bilateral vestibulopathy; in a special subgroup of them, those with profound hearing loss, the treatment challenges are extreme. Electrical stimulation of the saccular afferents is one of the solutions provided to them, and Curthoys et al. have provided data of improved performance. They also proposed an interesting theory to connect this type of stimulation to nonvestibular brain areas that could potentially be of benefit for other gait disorders, such as that which occurs in Parkinson’s disease [2].

The importance of correct and complete anamnesis in patients with vestibular disorders has been stressed many times. Van de Berg and Kingma [3] have provided a simple to follow and systematic approach for nonacute symptoms that are of major concern on a daily basis to clinicians, pointing to a step-by-step consideration. They call attention to the possibility of different disorders that could occur metachronically or synchronically, as respectively shown in the next two papers. First, Oka et al. give an interesting functional explanation of the findings in patients with persistent postural-perceptual dizziness that exemplifies the need for thorough clinical evaluation and functional examination: considering previous disorders would enable a better understanding of current problems [4]. Second, the coincidence of two very distinct disorders—a very common one, benign paroxysmal positional vertigo (BPPV), and a very unusual one, bilateral vestibulopathy—has been systematically evaluated for the first time by Pérez-Fernández et al., who rely on, and strongly recommend, complete vestibular examination regardless of a differential diagnosis, and raise the question as to what the limit of vestibular function is for a nystagmic response to appear [5]. Two other papers deal with debatable clinical problems, giving light to a better understanding of them. In the first, Gill-Lussier et al. carried out a scoping review of proprioceptive cervicogenic dizziness [6], showing how heterogeneous this concept is regarding etiology, differential diagnosis, measurement, and treatment, also providing a good characterization for further studies. In the second, Idriss et al. have shown the intriguing place of the narrow internal auditory canal in pediatric vestibular paroxysmia [7]; both are unusual conditions in the studied population, and the authors direct some remarks to the methodology of imaging to obtain an adequate result.

The vestibular evaluation of patients is always initiated with a bedside examination of vestibular reflexes and nystagmus. In this regard, Gufoni and Casani [8] have made an interesting observation and conducted clinical research to show that pupillary hippus is substantially associated with vestibular migraines, particularly during the interictal phase. Their methodology is so easy to follow as to become something to include in common daily bedside and instrumental examinations, deserving interest when other disorders, such as Ménière’s disease or positional vertigo, are coincident with migraines. The development of the video head impulse test (vHIT) since its characterization [9] is still ongoing. One of the first derivatives was the suppression head impulse (SHIMP) test: van Dooren et al. compare the head impulse (HIMP) and SHIMP tests using the video system to track eye movement velocity in patients with bilateral vestibulopathy [10] and show how both tests are well correlated, though the gain in the vestibulo-ocular reflex is lower in the SHIMP test (as has been shown in other disorders or even normal subjects), but without clinical relevance for the final diagnosis. One of the most important vestibular tests of nystagmus is that induced by caloric stimulation, with air or water, of the inner ear through the external auditory canal. The caloric test has been associated with residual symptoms that prevent its frequent and repetitive use; these can be reduced by simply reducing the number of stimulations. According to Alhabib and Saliba, the use of only warm stimulation could solve this problem without losing value, and they recommend proceeding in both ears in cases without spontaneous nystagmus, providing the correct methodology when it is present or the final results are extremely low [11]. One of the missions of the vestibulo-ocular reflex is to maintain visual acuity during active or passive movement of the subject; Rodriguez-Montesdeoca et al. have shown that the unilateral electrical stimulation of the sacculus (not directly involved in that mission) in patients with bilateral vestibulopathy partially restores dynamic visual acuity function [12], and they try to answer this new finding in the correction of oscillopsia. They have opened, as shown in [2], a new avenue of research on the otolithic system.

The treatment of vestibulopathy was one of the main purposes of this Special Issue, and five papers were devoted specifically to it; although, as previously shown, some solutions for others have previously been presented. First, Esteban-Sanchez and Martin-Sanz [13] have covered the topic of acute unilateral vestibulopathy in a rigorous and detailed study during a follow-up, trying to correlate measures of functional damage, postural deficiency, and disability to the clinical state of compensation, with clinical implications when vestibular rehabilitation is the treatment of choice. Induced bilateral vestibulopathy is the unwanted effect of some systemic medications, such as gentamicin; in their study, Ferreira-Cendón et al. have shown how this effect could be reduced in patients with infectious endocarditis when an otoneurologist is included in the treating team to provide careful and continued measures of vestibular function to help in the continuation, or not, of gentamicin [14]. This probably could be expanded to other treatments with potentially ototoxic medications. The treatment of BPPV has been an intense topic in the literature since the description by Epley in 1979 of the first particle repositioning maneuver, or PRM [15]. Lee et al. present a new therapeutic maneuver for horizontal canal cupulolithiasis, which explains some common and difficult cases of BPPV; being aware of the problems that extreme inertia movements or mastoid oscillation have, as used in commonly performed PRM, they present a method that avoids them and should be considered, given their results [16]. Intractable Ménière’s disease has been approached with the use of intratympanic medication (dexamethasone, gentamicin) or surgery; Bae et al. present their report on the combined use of both medications to obtain subablative damage with gentamicin and reduce the ototoxic damage inherent to the treatment [17]. This is performed surgically through easy access to the middle ear; it was proposed some time ago, but their results look promising if the treatment is considered first-line or as a rescue when intratympanic gentamicin fails to provide good control of the disease.

The well-known complex relationship between vestibular deficiency, disability, and handicap indicates how important it is to adequately cover all of them to obtain a better understanding of the disease and the limitations of patients. Wood et al. have examined that cases of traumatic brain injury (TBI) in military personnel [18]. They have found some differences with nonmilitary patients with TBI (data obtained through peer-reviewed articles) that indicate the need for specific evaluation and treatment in the former based on the specific demographic characteristics of the population under study and the major relevance of vestibular dysfunction. This is an interesting approach to expand in further sociodemographic studies.
